# Sense of coherence and quality of life in older in-hospital patients without cognitive impairment- a 12 month follow-up study

**DOI:** 10.1186/1471-244X-14-82

**Published:** 2014-03-19

**Authors:** Anne-Sofie Helvik, Knut Engedal, Geir Selbæk

**Affiliations:** 1 Department of Public Health and General Practice, Faculty of Medicine, Institutt for samfunns medisin, Norwegian University of Science and Technology (NTNU), Postboks 8905, Trondheim NO-7491, Norway; 2 Division Tynset, Innlandet Hospital Trust, Tynset, Norway; 3 St Olav’s University Hospital, Trondheim, Norway; 4 National Advisory Unit on Ageing and Health, Vestfold Hospital Trust, Tønsberg, Norway; 5 Centre for Old Age Psychiatric Research, Innlandet Hospital Trust, Ottestad, Norway; 6 Akershus University Hospital, Lørenskog, Norway

**Keywords:** Depressive symptoms, Older adults without cognitive impairment, Coping, Mental health, Salutogenetic Theory, Personal ADL

## Abstract

**Background:**

The relation between sense of coherence (SOC) and quality of life (QoL) among older persons has been found in some, but not all, studies and mostly in studies with cross-sectional design. We wanted to study if SOC was associated with domains of QoL at hospitalization and one year later among persons 65 years and above without cognitive impairment.

**Method:**

At hospitalization (T1) and 12 month follow-up (T2) QoL and cognitive status were assessed using the WHOQOL-BREF and the Mini-Mental State Examination. At baseline, the 13-item version of the SOC scale was used to assess coping, the Hospital Anxiety and Depression Scale (HADS) was used to assess depressive and anxiety symptoms. Level of functioning was rated using Lawton and Brody’s scales for physical self-maintenance and instrumental activities of daily living (personal and instrumental ADL).

**Results:**

In total, 165 (80 men) persons with a mean age of 77.7 (SD 6.9) years were included. The proportion of people rating their overall QoL as high had decreased from T1 to T2. The mean score on QoL- physical domain had increased, while the mean score of QoL-environmental domain had decreased. In adjusted regression analyses at T1, a high level of SOC was positively associated with QoL in three of four domains, i.e. physical, psychological and environmental, but level of SOC assessed at T1 was not associated with any domain of QoL at T2. Personal ADL was associated with some domains of QOL at T1 and T2.

**Conclusion:**

The SOC level was associated with older adult’s QoL during hospitalization but not their QoL one year after the hospital stay.

## Background

In planning of health care services the assessment of older adult’s quality of life (QoL) is important and can provide a clinical outcome measure of health care [1-3]. In line with the definition of QoL by the World Health Organization (WHO), the QoL is subjective and depends on individuals’ perception of their position in life [4]. It is a multidimensional concept covering the physical, psychological, social and environmental aspects of life [2,5], but is something different from symptoms, functional level and other regular health indicators [6].

Health may influence QoL without determining it [2] and physical and psychological health has to some extent been reported to be associated with QoL or domains of QoL in older patients with medical problems [7-17]. For example depression is common in older adults with chronic health problems and it is reported that their QoL decreases with an increasing severity of depression [18]. Consequently, research into how physical and psychological health influence, or are associated with QoL in older adults is increasing. Quite recently, some cross-sectional studies of QoL among a broad sample of older medically hospitalized older adults have been published [19,20]. These studies reported that low QoL was associated with depression and anxiety.

How you cope with internal and external stressors in life may have importance for QoL. Sense of coherence (SOC) is a way of seeing the world that facilitates successful coping with stressors [21]. Three core components capture the SOC concept: comprehensibility, manageability and meaningfulness [21-23]. These components describe to which degree the individual has a feeling that stimuli from internal or external environments are structured, explicable and predictable. Furthermore, they describe whether the individual has resources available to meet the demands from these stimuli and experiences the demands as challenges and worthy of engagement and investment [21]. A systematic review of SOC in 32 papers up to 2003 found that high SOC was related to better QoL [24], but with some exceptions. Three of the reviewed studies were performed among older adults. Since the SOC questionnaire is not easy to fill in [23,25], newer studies have included only older adults without cognitive impairment. In recent years the importance of SOC in relation to QoL have been studied in cross-sectional studies among older adults [26-28], in older adults suffering from somatic difficulties staying at home [29-33] or in older adults being hospitalized [34] and, lastly, among older adults in nursing homes [35]. An association between high SOC and higher levels of overall QoL or domains of QoL assessed in cross-sectional studies were reported in most, but not in all studies [26,27]. More specifically, most cross-sectional studies of older people reported that a high SOC was related to high QoL, but in the oldest old (85 years and above) people and in older persons with cancer a high SOC was not related to QoL [26,27].

According to Antonovsky’s theory, it is reasonable to expect that older people with a high SOC may experience a high QoL, also over time, even if they might be susceptible to reduced health state, reduced daily living capacity and changes in their life situation over time due to their increasing age. It is suggested that older adults with a high SOC are more likely to take an active role in shaping their own future outcome, i.e. a high SOC has relevance for a high QoL in the future [31]. On a theoretical basis, Antonovsky argues that SOC is relatively stable after the age of 30, but others have found SOC less stable than expected [23,36,37] and to our knowledge relatively few studies have explored how SOC and health related variables at one time point may have importance for QoL or domains of QoL in the future.

Of the large variety of QoL inventories used in research studies involving older individuals [38,39], a commonly used multidimensional QoL inventory in older adults is SF-36 [7,39,40]. A more recent inventory, developed across cultures is the relatively short multidimensional WHOQOL-BREF (26 items) which is adequate for older adults [41], satisfies our definition of the QoL [3,42], is designed to be used for both seriously ill and repeated assessment [42,43] and appropriate for exploring health indicators such as physical functional status, and coping resources are related to domains of QoL [44,45].

The main purpose was to study, in a prospective study, if SOC was associated with QoL among older persons with somatic health problems. In order to do so we wanted to study QoL assessed by the WHOQOL-BREF at hospitalization (T1) and one year after (T2) in older adults (≥65 years) without cognitive impairment at baseline, and to explore factors associated with physical, psychological, social and environmental domains of QoL at both times with a particular focus on if and how SOC was associated with QoL. We hypothesized that high SOC and good physical and/or psychological health at T1 would be associated with better QoL both at T1 and T2.

## Methods

During a two-year period (1 September 2006 – 30 August 2008) older adult patients (≥ 65 years) at a general public hospital in Norway [20,46] were included in this longitudinal study. The hospital serves nine rural municipalities, covering an area of 15,000 km^2^ inland with 25,000 inhabitants, where 4,600 persons are 65 years or older. The main admitting diagnoses to the hospital were cardiovascular and/or pulmonary diseases [20,46].

The participants were followed up one year after inclusion. The same two research nurses (one specialized in geriatrics and one in health science) collected the data at both points in time. Prior to the start of the study, the nurses completed a two-day course on how to conduct the interview, followed by practicing on a number of healthy subjects. The inter-rater reliability between the two nurses was checked at baseline for the 30 first study participants and found acceptable (the Spearman’s rho varied from 0.91-0.97).

All the patients aged 65 years or older were invited to participate during their hospital stay just after they had been medically stabilized. The date and time of their inclusion in the study was registered. The patients received written and verbal information about the study, and they subsequently gave their written consent. Initially, the Mini-Mental State Examination (MMSE) [47] and subsequently the Clinical Dementia Rating Scale (CDR) [48] were administered to all potential patients to rate the severity of cognitive impairment. Assessment of SOC was conducted in patients with minimal or no cognitive impairment (MMSE > 24). If needed, the patients were given help to read and tick off the self-report questionnaires.

After one year (± 14 days), the patients were followed-up (T2) and the MMSE, CDR and WHOQOL assessments were repeated.

The study was approved by the Regional Committee for Medical Research Ethics in South-Eastern Norway and the Norwegian Social Science Data Service.

### Participants

All patients 65 years and older, living in the region, admitted to the internal medical inpatients service of the Tynset Division of the Innlandet Hospital Trust with an acute medical condition, and hospitalized for at least 48 hours were potential participants. Of the 802 possible study participants, 318 (40%) were excluded due to: severe cognitive impairment (116 patients) signified by a score of three on the CDR [48,49]; severe communication difficulties mainly caused by profound speech difficulties and severe hearing loss (25 patients); being in a terminal state or having died before inclusion (47 patients); reduced physical functioning that made completion of the protocol impossible (mainly caused by severe cardiovascular, pulmonary or cancer diseases) (106 patients); or, refusal to participate (24 patients) [20,46]. Thus, 484 patients were assessed for inclusion in this study. Among these, 267 patients were excluded due to a score of 24 or lower on the MMSE [47], indicating significant cognitive impairment [50]. Thus, 217 participants were assessed at baseline (T1).

At follow-up (T2), 27 had died, 7 participants had a score of three on the CDR indicating severe cognitive impairment, and 18 declined to participate. In total, 165 (76.0%) patients participated in the follow-up study.

### Measures

*The quality of life* was measured twice (T1 and T2) with WHOQOL-BREF, a second generation QoL questionnaire produced by the World Health Organization (WHO), consisting of 26 items. The items are grouped into four domains; the physical (7 items), the psychological (6 items), the social (3 items) and the environmental (8 items), together with 2 items of a global character, i.e. overall QoL and general health [43,51]. Each item is answered on a 5-point response scale, and the three negatively formulated items have a reversed scale. Thus, higher scores indicate a better QoL. The score on each domain is found by multiplying the calculated mean value of the items belonging to the domain by four giving scores ranging from 4 to 20 [42,43,51]. The two items of global character, overall QoL and general health scores, were dichotomized; i.e. high (score 4 and 5) vs. not (score 1–3) high [52]. The WHOQOL-BREF domains correlate well with WHOQOL-100 and results from such studies may be directly compared [4,43,53]. The questionnaire has been translated into Norwegian and validated [54] and used with various categories of patients, including older adults [52].

*Sense of coherence* was assessed at T1 with the 13-item version of the sense of coherence questionnaire (SOC) [55]. The five negatively formulated items were reversed and the sum score of the questionnaire ranged from 13 to 91. A higher SOC score indicates better capacity to respond adequately to stressful situations [56]. Previously, the SOC questionnaire has been used among older subjects with minimal, but not significant, cognitive impairment [33-35,57-59]. The questionnaire has been translated into Norwegian and used in several studies in Norway [55,56,60].

*Physical health* (number of hospitalizations in the previous five years, length of hospital stay, and diagnoses at inclusion in the study) was obtained at T1 from medical records or hospital administrative systems. Details of co-morbid diseases were collected at T1 using the Charlson Index [61] and employing Schneeweiss weighting [62]. A higher score indicates more severe comorbidity.

*Level of functioning* (Activities of Daily Living - ADL) was measured at T1 by the Physical Self-Maintenance Scale (PSMS, score range 6–30) and the instrumental activities of daily living scale (I-ADL, score range 8–31) [63]. In this paper we report the score of the PSMS scale as a “Personal ADL” measure. A higher score on both ADL scales indicates a poorer functioning [63].

*Cognitive function* was assessed twice (T1 and T2) by means of the Mini-Mental State Examination (MMSE), a 30-point questionnaire [47]. A MMSE score higher than 24 indicates minimal or no cognitive impairment [50]. The Clinical Dementia Rating Scale (CDR) assessed the severity of dementia and a total score of 3 (range 0–3) indicates severe dementia [48].

*Depressive and anxiety symptoms* were assessed at T1 using the self-report inventory Hospital Anxiety and Depression Scale (HADS). The HADS has 14 items assessing depressive and anxiety symptoms (seven items each with a score range of 0–21) [64]. High scores indicate more severe symptoms. The cut-off point for having clinically significant depression (HADS-D score) or anxiety (HADS-A score) was set to ≥ 8 in each sub-scale [65]. The HADS has been translated into Norwegian and validated in Norway and used in several studies including some among older adults [46,66].

*Socio-demographic information* (living alone or not, smoking habits and residence details) was self-reported using questions from the population-based health studies undertaken in Nord-Trøndelag county [67].

### Data analysis

Data were analyzed by means of the PASW, version 18.0 (Chicago, Ill, USA). Descriptive analysis of independent samples was performed with the chi-square statistic or Fisher’s Exact Test for categorical variables (depending on the number of cases included). Independent samples *t*-tests/ANOVA or the nonparametric Mann–Whitney test was applied for continuous variables (depending on whether or not the distribution was normal). A comparison of individuals and the domains of QoL at T1 and T2 were made by means of tests for dependent samples; i.e., dependent sample *t*-tests, or the Wilcoxon Signed Rank test for continuous variables (depending on whether or not the distribution was normal).

The outcomes, domains of QoL at T1 and T2 were normally distributed and analyzed by univariate and multiple linear regression (the ‘Enter’ method). Socio-demographic and health-related variables, as well as SOC at T1 were seen as independent variables [6]. In the initial unadjusted models of domains of QoL, the importance of gender, age, living status, Charlson Index, MMSE, personal ADL and I-ADL, HADS-D, HADS-A and SOC at T1, were explored. Furthermore, domains of QoL at T2 were explored using the same independent variables as well as new hospitalization since T1 (yes/no) and additionally adjusted for the score of the actual domain of QoL at T1. The lowest score level was set as the reference level when possible. In the presented adjusted models of QoL at T1 and T2, variables with p-value ≤ 0.1 in the primary linear regression analysis of one of the domains of QoL were included in the adjusted analyses.

The analyses were checked for interactions and p-values ≤ 0.05 were regarded as statistically significant.

## Results

### Sample characteristics

In all, 80 men and 85 women participated at both the baseline (T1) and follow-up (T2) examination (see Table 1 for characteristics of the participants). At T1 the patients’ age range was 65–98 years (mean 77.7, SD 6.9 years).

**Table 1:** Characteristics of study sample by level of SOC at baseline (n = 165)

		**Low SOC**		**Medium high SOC**		**High SOC**		**Total**	
*Socio-demographic*									
Women	N (%)	24	*(42.86)*	36	*(61.02)*	25	*(50.00)*	*85*	*(51.52)*
Age (by year)	Mean *(SD)*	76.73	*(7.25)*	78.64	*(7.35)*	77.76	*(6.00)*	77.73	*(6.93)*
Smoking	N (%)	8	*(14.29)*	5	*(8.47)*	7	*(14.00)*	*20*	*(12.12)*
Living alone	N (%)	32	*(57.14)*	32	*(54.24)*	23	*(46.00)*	*87*	*(52.73)*
*Information on physical health*									
Previous hospitalisations last 5 years	Mean *(SD)*	1.96	*(2.40)*	1.98	*(2.40)*	1.94	*(2.71)*	1.96	*(2.48)*
Actual hospitalisation (days)									
Duration of hospital stay	Mean *(SD)*	5.41	*(3.90)*	5.76	*(3.92)*	5.36	*(3.92)*	5.52	*(3.88)*
Duration before inclusion	Mean *(SD)*	2.75	*(1.73)*	4.02	*(2.78)*	4.22	*(3.33)*	3.65	(2.73)*
Charlson Index	Mean *(SD)*	1.84	*(1.62)*	2.27	*(2.32)*	1.56	*(1.46)*	1.91	*(1.87)*
*Functional capability*									
P-ADL	Mean *(SD)*	7.25	*(1.76)*	7.36	*(1.63)*	7.26	*(2.21)*	7.29	*(1.85)*
I-ADL	Mean *(SD)*	7.46	*(2.86)*	8.09	*(2.45)*	8.44	*(2.01)*	7.98	*(2.50)*
*Anxiety and depression*									
Depression (HADS-D score ≥ 8)	N (%)	6	*(10.71)*	1	*(1.69)*	2	*(4.00)*	*9*	*(5.45)*
Anxiety (HADS-A score ≥ 8)	N (%)	9	*(16.07)*	3	*(5.08)*	1	*(2.00)*	*13*	*(7.88)*

*SOC* = Sense of Coherence, *P-ADL* = Personal all day living function, *IADL* = Instrumental all day living function, *HADS-D* = Depressive symptoms of the Hospital Anxiety and Depression Scale, *HADS-A* = Anxiety symptoms of the Hospital Anxiety and Depression Scale.

*p ≤ 0.05 analyzed with ANOVA by level of SOC.

The 52 persons who did not participate at T2 had significantly poorer personal ADL (mean 8.2, SD 2.3 vs. mean 7.3, SD 1.9; p < 0.05), and had more often nursing assistance in daily life activities at T1 (31.4% vs. 16.4% p < 0.05). However, the age and gender distribution, the prevalence of anxiety and depressive symptoms and the level of QoL of the individual domains were similar among those who completed T2 and those who dropped out prior to T2. The mean follow-up time was 370 (SD 29.3) days.

At T2 compared with T1, a higher proportion of the participants had assisted living (34.5% vs. 27.3%; p < 0.05) and they were more cognitively impaired (MMSE score: mean 25.2, SD 3.1 vs. mean 27.6, SD 1.6; p < 0.01). In all, 68 (41.2%) had an MMSE score ≤ 24 at T2. Furthermore, 68 (41.2%) of the participants had been re-hospitalized between T1 and T2.

### Quality of life

The overall QoL at T1 was high in more than three quarters (136/165, 82.4%) of the participants and the mean domain scores of WHQOL-BREF varied from 12.6 (physical) to 15.3 (environmental) (Table 2). From T1 to T2, the proportion of older adults assessing their overall QoL as high had decreased (82.4% vs. 120/165, 72.7%; p < 0.05). The mean score of the physical domain of QoL had increased from T1 to T2 (mean score 12.6, SD 2.5 vs. mean score 13.4, SD 1.97; p < 0.01), while the mean score of the environmental domain of QoL had decreased (mean score 15.3, SD 1.6 vs. mean score 13.2, SD 1.2; p < 0.01). The mean score of the psychological and social domains of QoL were unchanged (see Table 2).

**Table 2:** **Descriptive characteristics of domains of QOL at baseline (T1) and one year after (T2) (N = 165)**^**1**^

	**QoL at T1**								**QoL at T2**							
	**Physical**		**Psychological**		**Social**		**Environmental**		**Physical**		**Psychological**		**Social**		**Environmental**	
***SOC at T1***	**Mean *(SD)***		**Mean *(SD)***		**Mean *(SD)***		**Mean *(SD)***		**Mean *(SD)***		**Mean *(SD)***		**Mean *(SD)***		**Mean *(SD)***	
Low	11.51	*(2.67)*	14.56	*(1.62)*	14.13	*(1.79)*	14.81	*(1.68)*	13.00	(2.09)	14.86	(1.69)	14.26	(1.50)	12.93	(1.42)
Medium high	12.89	*(2.17)*	15.71	*(1.48)*	14.68	*(1.64)*	15.46	*(1.76)*	13.23	(1.92)	14.90	(1.28)	14.58	(1.26)	13.15	(1.25)
High	13.49	*(2.10)*	15.72	*(0.99)*	14.71	*(0.81)*	15.67	*(1.23)*	14.18	(1.68)	15.60	(1.14)	14.72	(0.54)	13.46	(0.92)
*Total*	12.61	(2.45)**	15.35	(1.49)**	14.50	*(1.51)*	15.31	(1.62)*	13.44	(1.97)**	15.09	(1.43)*	14.51	(1.20)	13.17	(1.23)

*QoL* = Quality of life, *SOC* = Sense of Coherence.

*p ≤ 0.05, **p ≤ 0.01 analyzed with ANOVA by level of SOC.

^1^A higher score indicate higher QoL (possible score range are 4–20).

### Factors associated with quality of life at T1 and T2

In the multiple regression models of the QoL domains at T1 independent health-related variables and SOC were adjusted for each other and for socio-demographic factors (see Table 3). In adjusted regression analyses at T1, medium high and high degrees of SOC were positively associated with QoL in three of four domains, i.e. physical, psychological and environmental. In addition, at T1, high personal ADL functioning was associated with increased physical and environmental QoL; low depressive symptom score (HADS-D < 8) was associated with increased physical and psychological QoL; and low anxiety symptom score (HADS-A < 8) was associated with increased psychological QoL.

**Table 3:** Multiple linear regression analysis of the WHOQOL-BREF domains at T1 and T2

	**Domains of QoL at T1**						**Domains of QoL at T2**					
	***β***_***1***_	**(95% CI)**		***β***_***2***_	**(95% CI)**		***β***_***3***_	**(95% CI)**		***β***_***4***_	**(95% CI)**	
***Physical QoL***												
SOC Medium high	1.43	0.55	2.31**	1.31	0.47	2.15**	-0.12	-0.82	0.59	-0.04	-0.75	0.67
High	1.20	1.09	2.90**	1.76	0.89	2.62**	0.66	-0.08	1.41	0.65	-0.10	1.40
P-ADL > 6				-1.60	-2.30	-0.89**				-0.76	-1.38	-0.14*
HADS-D ≥ 8				-1.80	-3.34	-0.26*				1.07	-0.21	2.36
HADS-A ≥ 8				0.02	-1.29	1.34				-0.71	-1.79	0.37
Adjusted R^2^		10.0			21.8			13.2			13.2	
***Psychological QoL***												
SOC Medium high	1.16	0.63	1.70**	0.92	0.39	1.45**	-0.29	-0.83	0.25	-0.32	-0.85	0.,22
High	1.16	0.61	1.72**	0.91	0.37	1.46**	0.37	-0.20	0.93	0.30	-0.26	0.85
P-ADL > 6				-0.16	-0.60	0.28				-0.51	-0.95	-0.08*
HADS-D ≥ 8				-1.33	-2.29	-0.37**				-0.50	-1.46	0.46
HADS-A ≥ 8				-0.99	-1.84	-0.15*				-0.53	-1.36	0.31
Adjusted R^2^		11.4			21.8			11.3			14.7	
***Social QoL***												
SOC Medium high	0.52	-0.05	1.09	0.46	-0.12	1.04	0.20	-0.25	0.64	0.18	-0.17	0.76
High	0.57	-0.02	1.15	0.49	-0.11	1.08	0.34	-0.12	0.80	0.30	-0.74	0.72
P-ADL > 6				-0.32	-0.80	0.17				-0.32	-0.70	0.07
HADS-D ≥ 8				-0.64	-1.70	0.43				-0.30	-0.70	0.07
HADS-A ≥ 8				-0.03	-0.97	0.91				-0.01	-1.13	0.54
Adjusted R^2^		1.3 N.s. model			1.6 N.s. model			4.9			5.2	
***Environmental QoL***												
SOC Medium high	0.87	0.30	1.57	0.77	0.23	1.30**	-0.04	-0.37	0.30	-0.05	-0.39	0.29
High	0.96	0.37	1.55	0.73	0.18	1.27**	0.13	-0.22	0.48	0.10	-0.25	0.45
P-ADL > 6				-1.29	-1.73	-0.84**				-0.25	-0.56	0.06
HADS-D ≥ 8		12.1		-0.57	-1.55	0.41				-0.38	-0.10	0.24
HADS-A ≥ 8				-0.66	-1.52	0.20				-0.23	-0.78	0.31
Adjusted R^2^		12.1			28.4			50.7			51.4	

*T1* = baseline, *T2* = one-year after, *QoL* = Quality of life, *SOC* = Sense of Coherence, *P-ADL* = personal all day living function, *HADS-D* = Depressive symptoms of the Hospital Anxiety and Depression Scale, *HADS-A* = Anxiety symptoms of the Hospital Anxiety and Depression Scale.

Independent variables were assessed at T1.

_*1*_Adjusted for age and gender.

_*2*_Adjusted for age and gender and the other variables included.

_3_Adjusted for age, gender and QoL of the actual domain at T1.

_4_Adjusted for age, gender, QoL of the actual domain at T1and the other variables included.

Adjusted R^2^ = explained adjusted variance.

N.s. Not significant.

*p ≤ 0.05, **p ≤ 0.01.

In analyses of QoL at T2 with adjustment of the same variables as for QoL at T1 and with adjustment for the level of the corresponding domain of QoL at T1, level of SOC at T1 was not associated with any domain of QoL (see Table 3). Personal ADL functioning was the only health-related variable from T1 associated with domains of QoL at T2, i.e. high personal ADL functioning was associated with increased physical and psychological QoL score at T2.

## Discussion

To the best of our knowledge, this is the first longitudinal study in Scandinavia that has examined domains of QoL at baseline (T1) and one-year follow-up (T2) in relation to level of SOC and health assessed at T1 among older medically hospitalized adults without cognitive impairment. The older adults with medium high and high SOC at T1 had higher physical, psychological and environmental QoL than those with low SOC at T1, also in analyses adjusted for physical functioning and mental health. However, level of SOC at T1 was not of importance for any domain of QoL one year later (T2). High personal ADL functioning assessed at T1 was the only health variable associated with high physical and psychological QoL at T2.

Comparing domains of QoL at hospitalization and one year after may give more nuanced information than studying the overall QoL. The mean score of the physical QoL domain in the participants was significantly increased from T1 to T2, while the environmental QoL domain was reduced. The mean score of the psychological and social domains of QoL remained relatively unchanged from T1 to T2, which is in line with the results reported in the Norwegian general population of young and older adults [44,54]. However, the mean scores of the physical and environmental QoL at T2 were lower than in Norwegian general populations [44,54]. This finding is difficult to interpret, but, we suggest that the group of older adults included in the present study may be more physically limited and living in a rural area may have consequences for the environmental QoL. The structure of the rural communities may contribute to additional challenges to reach health services, to use transportation and participate in community activities that are important for the environmental domain of QoL.

The QoL domains one year after hospitalization could not be explained by the level of SOC or psychological symptoms assessed at hospitalization. Based on Antonovsky’s theory [21,22], it was reasonable to expect that those with a high SOC at hospitalization were more likely to experience a high QoL one year after. However, the SOC does not seem to be as stable as suggested by Antonovsky [23]. The SOC may have been influenced by life events during one year [68,69], which we unfortunately were not able to adjust for. Even so, it is reasonable to expect that the SOC level assessed at hospitalization would be of less importance one year later since having a high age and being vulnerable to further health deterioration may be linked to several negative life events in the year before follow-up. The SOC level assessed at hospitalization may have changed after hospitalization [36].

Health may influence QOL [2]. In line with previous cross-sectional studies among older persons we found depressive symptoms and personal ADL in older persons with medical problems associated with lower QoL [9,12,17-20]. In line with our study, a six month follow-up study of women with myocardial infarction reported personal ADL important for the physical and psychological domains of QOL at follow-up [33]. However, of the variables describing the health situation at hospitalization only the personal ADL influenced the QoL one year after discharge from the hospital, which is in line with a review finding the health variables to have less importance for QoL over time in older persons compared to middle-aged persons [7].

The study has some limitations. Firstly, the number of participants was restricted due to the presumption of adequate cognitive function (MMSE > 24) at baseline in order to assess SOC. This may be a high threshold since there does not seem to be an exact limit for acceptable cognitive function in the use of SOC questionnaires [56,60]. To increase the number of participants we could have used persons well known to the patients as proxy informants. This approach might have led to new methodological problems; e.g. previous research has reported low correlation between QoL rated by the patient themselves and a proxy person [70]. The MMSE is a screening tool to assess cognitive function, not a diagnostic tool, thus, even if we used the recommended cut-off used for Norwegian samples [50], we cannot eliminate the risk of having included participants with minimal cognitive impairment or even dementia [71-73].

Secondly, at T2 several of the participants (41.2%) had reduced cognitive functioning (MMSE ≤ 24). This indicates that the studied sample was a vulnerable group of older adults. The criteria set for assessing SOC was not fulfilled in nearly half of the participants at T2. Including the SOC at T2, which is interesting since stability of SOC is questioned in older persons, would have reduced the sample size dramatically and reduced the ability to answer the research question. In addition, one may question whether these participants with reduced cognitive function at follow-up could give valid responses to the HADS and the WHOQOL-BREF. The HADS has been recommended for screening of depressive symptoms in medically hospitalized patients [64,74,75]. Furthermore, in a newly published study including a sample of patients with reduced cognitive function, comparable to our sample with regard to cognitive impairment, the validity of the HADS-D was reported to be good [76]. The sensitivity and specificity of the HADS-D with a cut-off 7/8 was 0.92 and 0.87, respectively [76]. In addition, the QOL-inventory has good psychometric properties in older adults with mild and moderate dementia [77].

Thirdly, SOC was assessed with the 13-item version of the questionnaire. We would argue that this should not be a problem since studies confirm that the 13-item version can be substituted for the original 29-item version [21,22,78]. Furthermore, categorizing the SOC variable at baseline using the 33.3 and 66.6 percentile to define the boundaries for levels of SOC may be questioned. Antonovsky talks in general terms about high and low SOC but he never defined cut-offs for low, medium high or high scores for SOC [21,22]. Even so, the categorization done in the present study is in line with the categorization of the SOC variable frequently performed by others [55,56].

## Conclusion

SOC was associated with QoL at hospitalization, but not at 12-month follow-up. Thus, in hospital practice, the SOC level seems less suitable for predicting future QoL in older adults. The most probable explanation is that SOC is not as stable as first assumed in the Salutogenetic Theory. The personal ADL at hospitalization may influence the QoL up to one year after discharge from the hospital.

### Relevance for clinical practice

Since the patents’ personal ADL at hospitalization was independently associated with QoL not only at hospitalization, but also up to one year after discharge, the quality of the nursing care given during hospitalization to strengthen the personal ADL of the older patient may have vital importance for them. Thus, nurses need to focus on improvement of Personal ADL in older persons with somatic health difficulties in order to increase their QoL.

## Competing interest

The authors declare that they have no competing interests.

## Authors’ contribution

ASH was responsible for developing the research idea, the data collection and performing the statistical analysis. KE and GS have participated in the design and the analysis, and all authors participated in the preparation of the manuscript. All authors read and approved the final manuscript.

## Pre-publication history

The pre-publication history for this paper can be accessed here:

http://www.biomedcentral.com/1471-244X/14/82/prepub
